# Data on motivational factors of the medical and nursing staff of a Greek Public Regional General Hospital during the economic crisis

**DOI:** 10.1016/j.dib.2017.02.026

**Published:** 2017-02-16

**Authors:** Marianna Charalambous, Mitosis Konstantinos, Michael A. Talias

**Affiliations:** a Open University of Cyprus, P.O. Box 12794, Cyprus; b National Resistance 21, P.O. Box 64100, Eleftheroupoli Kavala; c Faculty of Economics and Management, Open University of Cyprus, Cyprus

**Keywords:** Motivational factors, Economic crisis, Medical and nursing staff, Public health, Multilevel Rasch Model Greece

## Abstract

In this article, we present the data related to motivational factors given by the medical (n=118) and nursing (n=217) staff, of a Greek Public General Hospital during a period of financial austerity. The data collection has been based on a structured self-administrable questionnaire which was used in a previous survey in Cyprus (Chatzicharalambous, 2015) [1]. The incentives-rewards included amount in a total to 11 (both financial and non-financial). The data contains 4 parts: (1) demographics, (2) assessment of the degree to which this hospital provided such incentives-rewards, (3) personal assessment of the participants about the significance of these incentive-rewards and (4) to what extent these incentives-rewards have increased or decreased over the last five years due to the economic crisis. The sample was analyzed as a whole on demographics and by a professional subgroup (doctors and nurses) for the other three parts. The data include quantitative tables for all parts. Finally include three tables contain multilevel models.

**Specifications Table**

**Table:** 

Subject area	Health care management, Psychology
More specific subject area	Motivational factors
Type of data	Tables
How data were obtained	The sample collected using a structured self-administrable (Chatzicharalambous, 2015) [1].
	Data were collected from the medical and nursing staff from all sectors, independent specialty (dentist, surgery etc.), age and gender.
Data format	Analyzed
Experimental factors	A pilot study was conducted by using the questionnaire that was distributed to 10 nurses and 10 doctors who were asked to tell their opinion about how easy and understandable the questionnaire was and what adjustments they would propose to the research team to improve it.
Experimental features	The two categories of workers, nurses and doctors, tend to answer questions in a significantly different ways and we therefore use a multilevel Rasch approach to capture differences between and within levels and make comparisons between subgroups in the data. The Rasch models are estimated using the SIRT package of the open source statistical computing programming language R.
Data source location	General Hospital of Kavala, Kavala, Greece
Data accessibility	The data are available with this article

**Value of the data**

• The data present the motivational factors in medical and nursing staff of a general hospital in times of economic austerity.
• The data could be generalized to other hospitals of this type.
• This data may allow to other researchers to conduct a comparative evaluation with similar studies during the economic crisis.
• This data can be employed by other researchers to realize analyses based on different demographics.

## 1 Data

The dataset of this article provides information on the motivational factors of health professionals (doctors and nurses) in times of economic crisis. The survey is quantitative. Table 1 shows the demographic characteristic of the sample (n=335). Table 2, Table 3, Table 4 show the findings of the personal assessment of staff [doctors (n=118), nurses (n=217)] on motivational factors. More specifically, Table 2 contains the assessment of the degree to which this hospital provided such incentives-rewards. Table 3 contains the assessment extent of the significance level of incentives-rewards and Table 4 shows the assessment extent to which incentives-rewards over the past five years have changed (increased or decreased). Finally, we model our data using a multilevel item (package in R) response theory models. Tables 5, 6 and 7 contain the three models. Table 5 contains a model without intercept variances and no slopes. Table 7 contains a model with item wise intercept variance and no slope. Table 7 contains model with intercept variance and slope variances with hierarchical item and slope parameters.

## 2 Experimental design, materials and methods

### 2.1 Participants

The sampling was performed over a period of two months from July to August 2016. A total of 335 questionnaires were collected. Data were randomly collected from the medical and nursing staff from all sectors, regardless participants’ specialty (dentist, surgery etc.), age and gender. The 118 (35.2%) questionnaires came from the medical staff and the 217 (64.8%) from nursing staff.

### 2.2 Ethnics

The Hospital׳s health professionals (doctors and nurses) were informed of the content, purpose and that data would be used anonymously and only for the completion of the research. Their participation was voluntary. Along with the questionnaire, a cover letter given to staff, explaining the purpose and content of research. Moreover, for the conduct of the research and the distribution of the questionnaire, we sought permission from the administration of the hospital and the 4th Health Region which falls under the administration of the same hospital.

### 2.3 Questionnaire

To select the sample, we used a structured self-administrable questionnaire, which was used in a similar research by the Open University of Cyprus to the Limassol General Hospital [1].

We first conducted a pilot study by using the questionnaire which we distributed to 10 nurses and 10 doctors; they were asked to tell how easy and understandable the questionnaire was and what adjustments they would propose to the research team to improve it. The questionnaire consists of 4 parts and contains 40 questions in total. The first part contains 7 questions on demographics (*gender, age, educational level, marital status, specialty, years of service in the same hospital, years of total work experience*). The second part includes 11 questions about the incentives-rewards offered to the staff from the same hospital. The third part consists of 11 questions relating to the personal assessment of employees on how important they consider the incentives-rewards. The fourth and final part includes 11 questions related to the increase or reduction of employees rewards in the last five years due to the economic crisis. The 11 incentives-rewards which included was *salary, job security feeling, ability development (promotion), autonomy/feature initiative, developing skills and knowledge, good relationships with colleagues, good relationships with the manager, interested job object, equal treatment, good working conditions, recognition and bit estimate*. In the second and third parts, questions were based on a five point Likert-type scale (*none, little, enough, much, very much*). In the fourth part, the answers were also given on a five point Likert-type scale type (*falling too much, have fallen slightly, neither have declined nor have grown, have slightly increased, have grown too*). See Appendix.

### 2.4 Statistical treatment

We used the Statistical Package for Social Sciences (SPSS) for Windows (version 23.0, SPSS Inc. 2015) to analyze and elaborate the sample. Statistical significance level was set at *p*<0.05. Descriptive and inferential statistics was used to assess the responses to the questionnaire items. The sample was analyzed as a whole on demographics and by professional subgroups (doctors and nurses) for the other three parts. We created also a table for the demographics data. Findings of the other three parts were presented separately in tables as a percentage (%) for each subgroup.

### 2.5 Modeling data

We model our data using a multilevel response items modelling approach. This allow us to conceptualize better the variation of between and within groups of people and items and the people or items differences themselves at all levels with regard to the parameters of item response model. There are several packages in R for items response theory models. We use the SIRT package (Supplementary Item Response Theory Models) by Alexander Robitzsch which is freely available https://cran.r-project.org/web/packages/sirt/*,* CRAN version 1.13-1 (2016-11-17).

We use its function mcmc.2pno.ml which enable us to run the multilevel models of the 2 group for polytomous items assuming a Normal Multilevel Model. This function enables the estimation of random item models and multilevel IRT models using mcmc technique. The ability is decomposed into a Level 1 and Level 2.

The first model has no intercept variances and no slopes. We have 42 items and each beta denotes the difficulty parameter of each question. The second model has items parameters which are allowed to vary across groups. The third model has intercept variance and slope variances with hierarchical items and slope parameters. See Appendix A. Supplementary material.

## Funding sources

This research did not receive any specific grant from funding agencies in the public, commercial, or not-for-profit sectors.

## Figures and Tables

**Table 1:** Demographic characteristics of medical and nursing staff (N=335).

**Characteristics**	**N (%)**
**Gender**	
Man	104 (31.1)
Woman	231 (68.9)
	
**Age**	
20–30	31 (9.3)
31–40	122 (36.4)
41–50	135 (40.3)
51–60	43 (12.8)
60 and over	4 (1.8)
	
**Educational level**	
Diploma	28 (8.4)
Degree	254 (75.8)
Master	48 (14.3)
PhD	5 (1.5)
	
**Marital status**	
Unmarried	103 (30.7)
Married	192 (57.3)
Divorced	30 (9)
Other	10 (3)
	
**Number of children**	
0–2	262 (78.2)
3–5	67 (20)
6 and over	6 (1.8)
	
**Specialty**	
Doctor	118 (35.2)
Nurse	217 (64.8)
	
**Years of service in the same hospital**	
0–5	104 (31)
6–10	103 (30.7)
11–15	44 (12.5)
16–20	42 (12.5)
21–25	15 (4.6)
26-and over	27 (8.1)
	
**Years of total work experience**	
0–5	54 (16.1)
6–10	76 (22.7)
11–15	60 (18)
16–20	66 (19.7)
21–25	35 (10.4)
26 and over	44 (13.1)

Staff motivation in times of economic crisis.

**Table 2:** Assessment the degree to which this hospital provides the following rewards. Medical staff (N=118), Nursing staff (N=217).

	Medical staff (N=118)					Nursing staff (N=217)				
Motivational factors	None	Little	Enough	Much	Very much	None	Little	Enough	Much	Very much
	N(%)	N(%)	N(%)	N(%)	N(%)	N(%)	N(%)	N(%)	N(%)	N(%)
Salary	14(11.8)	44(37.2)	47(40)	10(8.5)	3(2.5)	12(5.5)	91(42)	74(34.1)	31(14.3)	9(4.1)
Job security feeling	14(11.8)	28(23.7)	55(46.6)	16(13.6)	5(4.3)	14(6.4)	56(26)	91(42)	48(22)	8(3.6)
Ability development (promotion)	7(6)	36(30.5)	55(46.6)	15(12.7)	5(4.2)	34(15.6)	60(27.6)	70(32.3)	38(17.5)	15(7)
Autonomy/Feature initiative	6(5.1)	28(23.7)	49(41.5)	28(23.7)	7(6)	11(5)	61(28.1)	71(32.7)	61(28.1)	13(6)
Developing skills and knowledge	7(5.9)	22(18.6)	60(51)	24(20.3)	5(4.2)	12(5.5)	57(26.2)	73(33.6)	58(26.7)	17(8)
Good relationships with colleagues	2(1.7)	11(9.3)	39(33)	52(44.2)	14(11.8)	1(0.5)	20(9.2)	70(32.2)	94(43.3)	32(14.8)
Good relationships with the manager	2(1.7)	8(6.8)	38(32.2)	50(42.3)	20(17)	1(0.5)	22(10.2)	57(26.2)	85(39.2)	52(23.9)
Interested job object	1(0.8)	11(9.3)	41(34.7)	52(44.1)	13(11.1)	4(1.8)	14(6.4)	69(31.8)	88(40.5)	42(19.5)
Equal Treatment	4(3.4)	12(10.2)	48(40.6)	50(42.4)	4(3.4)	10(4.6)	37(17)	66(30.4)	67(31)	37(17)
Good working conditions	4(3.4)	24(20.3)	67(56.8)	20(17)	3(2.5)	1(0.5)	39(18)	86(39.6)	64(29.5)	27(12.4)
Recognition and bid estimate	4(3.4)	32(27.2)	54(45.7)	24(20.3)	4(3.4)	18(8.3)	36(16.6)	86(39.6)	55(25.3)	22(10.2)

**Table 3:** Personal evaluation of the importance of rewards. Medical staff (N=118), Nursing staff (N=217).

	Medical staff (N=118)					Nursing staff (N=217)				
Motivational factors	None	Little	Enough	Much	Very much	None	Little	Enough	Much	Very much
	N(%)	N(%)	N(%)	N(%)	N(%)	N(%)	N(%)	N(%)	N(%)	N(%)
Salary	2(1.7)	9(7.6)	19(16.1)	48(40.7)	40(33.9)	4(1.8)	16(7.4)	19(8.8)	83(38.3)	95(43.7)
Job security feeling	1(0.8)	3(2.5)	15(12.7)	43(36.5)	56(47.5)	1(0.5)	14(6.4)	26(12.7)	68(31.4)	108(49)
Ability development (promotion)	3(2.5)	8(6.7)	16(13.6)	49(41.6)	42(35.6)	7(3.3)	11(5)	34(15.6)	83(38.3)	82(37.8)
Autonomy/Feature initiative	3(2.5)	3(2.5)	17(14.4)	52(44)	43(36.4)	3(1.4)	9(4.1)	35(16.1)	92(42.4)	78(36)
Developing skills and knowledge	1(0.8)	4(3.3)	10(8.5)	50(42.4)	53(45)	1(0.5)	7(3.3)	37(17)	90(41.4)	82(37.8)
Good relationships with colleagues	0(0)	0(0)	15(12.7)	53(45)	50(42.4)	0(0)	4(1.8)	25(11.5)	82(37.7)	106(49)
Good relationships with the manager	0(0)	5(4.2)	19(16.1)	44(37.3)	50(42.4)	0(0)	2(1)	29(13.4)	87(40)	99(45.6)
Interested job object	1(0.8)	2(1.7)	12(10.2)	52(44)	51(43.2)	0(0)	10(4.6)	15(7)	79(36.4)	113(52)
Equal Treatment	1(0.8)	2(1.7)	11(9.3)	53(45)	51(43.2)	3(1.4)	4(1.8)	25(11.5)	94(43.3)	91(42)
Good working conditions	0(0)	4(3.4)	13(11.3)	48(40.3)	53(45)	0(0)	6(2.7)	24(11)	80(37.3)	107(49)
Recognition and bid estimate	3(2.5)	3(2.5)	13(11)	51(43)	48(41)	3(1.4)	13(6)	25(11.5)	92(42.4)	84(38.7)

**Table 4:** Assessment the degree to which the rewards over the past five years have changed. Medical staff (N=118), Nursing staff (N=217).

	Medical staff (N=118)					Nursing staff (N=217)				
Motivation-al factors	Falling too much	Have fallen slightly	Neither have declined nor have grown	Have slightly increased	Have grown too	Falling too much	Have fallen slightly	Neither have declined nor have grown	Have slightly increased	Have grown too
	N(%)	N(%)	N(%)	N(%)	N(%)	N(%)	N(%)	N(%)	N(%)	N(%)
Salary	76(64.3)	27(23)	5(4.2)	3(2.5)	7(6)	146(67)	51(23.5)	13(6.3)	4(1.8)	3(1.4)
Job security feeling	46(39)	39(33)	22(18.6)	6(5.2)	5(4.2)	80(37)	71(32.7)	49(22.5)	14(6.4)	3(1.4)
Ability development (promotion)	23(19.4)	35(29.6)	47(40)	9(7.5)	4(3.4)	56(25.8)	71(32.7)	73(33.6)	16(7.4)	1(0.5)
Autonomy/Feature initiative	10(8.5)	36(30.5)	53(45)	16(13.5)	3(2.5)	27(12.7)	62(28.5)	97(44.6)	27(12.4)	4(1.8)
Developing skills and knowledge	44(37)	40(34)	13(11)	14(12)	7(6)	99(45.6)	59(27.2)	28(13)	24(11)	7(3.2)
Good relationships with colleagues	2(1.7)	27(22.9)	59(50)	23(19.4)	7(6)	9(4.3)	38(17.5)	126(58)	32(14.7)	12(5.5)
Good relationships with the manager	1(0.8)	24(20.5)	65(55)	13(11)	15(12.7)	9(4.3)	36(16.6)	123(56.6)	32(14.7)	17(7.8)
Interested job object	2(1.7)	20(17)	70(59.4)	23(19.4)	3(2.5)	8(3.6)	28(13)	124(57.1)	45(20.7)	12(5.5)
Equal treatment	2(1.7)	20(17)	73(61.8)	19(16.1)	4(3.4)	12(5.5)	48(22.2)	119(54.8)	30(13.8)	8(3.7)
Good working conditions	27(22.9)	42(35.6)	37(31.3)	8(6.8)	4(3.4)	83(38.2)	65(30)	35(16.2)	27(12.4)	7(3.2)
Recognition and bid estimate	10(8.5)	29(24.5)	67(56.9)	9(7.6)	3(2.5)	47(22.2)	112(51)	23(10.5)	28(13)	7(3.3)
