# Supplementary material for: Data on motivational factors of the medical and nursing staff of a Greek Public Regional General Hospital during the economic crisis
Source: Data Brief. 2017 Feb 16;11:371–81. doi: 10.1016/j.dib.2017.02.026 (PMC5331154; doi:10.1016/j.dib.2017.02.026)
Supplement: Supplementary file 1 — Supplementary material [file mmc1.docx]

No conflict.
