# Supplementary material for: Data on motivational factors of the medical and nursing staff of a Greek Public Regional General Hospital during the economic crisis
Source: Data Brief. 2017 Feb 16;11:371–81. doi: 10.1016/j.dib.2017.02.026 (PMC5331154; doi:10.1016/j.dib.2017.02.026)
Supplement: Supplementary file 2 — Supplementary material [file mmc2.zip › Model 2.docx]

Table 6

Model 2

Model with item wise intercept variance and no slope.

| Item Parameters |
| --- |
| parameter Mean SD MAP Rhat PercSEratio Q5 Q95 |
| 1 deviance 9294.975 15.884 9293.204 1.00 5.4 9268.513 9320.699 |
| 2 b[1] -1.503 0.047 -1.498 1.03 5.0 -1.578 -1.424 |
| 3 b[2] -1.643 0.053 -1.621 1.00 5.8 -1.736 -1.557 |
| 4 b[3] -2.620 0.072 -2.615 0.99 5.0 -2.739 -2.503 |
| 5 b[4] -2.129 0.055 -2.123 1.07 7.5 -2.218 -2.041 |
| 6 b[5] -1.819 0.058 -1.800 1.00 5.0 -1.917 -1.728 |
| 7 b[6] -1.228 0.052 -1.236 1.01 5.0 -1.313 -1.146 |
| 8 b[7] -1.498 0.047 -1.495 1.03 5.0 -1.576 -1.420 |
| 9 b[8] -2.403 0.101 -2.360 1.01 5.0 -2.566 -2.241 |
| 10 b[9] -3.150 0.109 -3.160 1.03 5.0 -3.329 -2.973 |
| 11 b[10] -1.608 0.021 -1.608 1.16 11.1 -1.643 -1.575 |
| 12 b[11] -1.823 0.021 -1.820 1.23 23.7 -1.856 -1.785 |
| 13 b[12] -1.754 0.026 -1.758 1.17 9.5 -1.794 -1.713 |
| 14 b[13] -2.015 0.022 -2.016 1.26 11.8 -2.053 -1.982 |
| 15 b[14] -2.016 0.023 -2.022 1.23 14.9 -2.053 -1.977 |
| 16 b[15] -2.586 0.021 -2.585 1.25 14.8 -2.619 -2.550 |
| 17 b[16] -2.708 0.019 -2.709 1.19 13.4 -2.740 -2.677 |
| 18 b[17] -2.617 0.020 -2.617 1.16 7.8 -2.648 -2.585 |
| 19 b[18] -2.353 0.022 -2.343 1.25 19.3 -2.390 -2.320 |
| 20 b[19] -2.149 0.021 -2.151 1.31 13.1 -2.183 -2.117 |
| 21 b[20] -2.025 0.022 -2.025 1.19 12.6 -2.060 -1.989 |
| 22 b[21] -3.057 0.024 -3.053 1.11 14.8 -3.098 -3.018 |
| 23 b[22] -3.247 0.024 -3.248 1.17 14.2 -3.286 -3.209 |
| 24 b[23] -3.012 0.025 -3.013 1.09 11.1 -3.053 -2.970 |
| 25 b[24] -3.079 0.020 -3.078 1.23 21.8 -3.115 -3.047 |
| 26 b[25] -3.196 0.020 -3.196 1.22 15.0 -3.228 -3.162 |
| 27 b[26] -3.312 0.021 -3.317 1.19 14.9 -3.346 -3.275 |
| 28 b[27] -3.238 0.021 -3.237 1.26 13.2 -3.273 -3.205 |
| 29 b[28] -3.313 0.022 -3.315 1.19 16.7 -3.350 -3.278 |
| 30 b[29] -3.250 0.021 -3.251 1.19 15.8 -3.285 -3.216 |
| 31 b[30] -3.296 0.022 -3.289 1.20 15.5 -3.330 -3.261 |
| 32 b[31] -3.138 0.022 -3.141 1.15 10.1 -3.174 -3.101 |
| 33 b[32] -0.546 0.037 -0.544 1.04 5.0 -0.606 -0.481 |
| 34 b[33] -1.024 0.030 -1.028 1.11 6.5 -1.075 -0.973 |
| 35 b[34] -1.347 0.023 -1.349 1.23 20.6 -1.383 -1.308 |
| 36 b[35] -1.668 0.022 -1.658 1.42 15.6 -1.702 -1.632 |
| 37 b[36] -1.071 0.037 -1.064 1.08 8.4 -1.131 -1.013 |
| 38 b[37] -2.020 0.022 -2.023 1.17 8.8 -2.057 -1.982 |
| 39 b[38] -2.097 0.023 -2.096 1.34 20.0 -2.133 -2.057 |
| 40 b[39] -2.076 0.021 -2.075 1.25 24.1 -2.110 -2.039 |
| 41 b[40] -1.951 0.022 -1.953 1.26 15.7 -1.989 -1.917 |
| 42 b[41] -1.243 0.033 -1.242 1.13 5.9 -1.297 -1.189 |
| 43 b[42] -1.475 0.029 -1.477 1.16 21.1 -1.524 -1.429 |
| 44 sigma1 0.805 0.032 0.814 1.00 5.0 0.754 0.857 |
| 45 sigma2 0.364 0.337 0.220 1.00 5.0 0.154 0.897 |
| 46 ICC 0.162 0.168 0.068 1.00 5.0 0.034 0.566 |
| 47 mu.b -2.221 0.135 -2.167 1.00 5.0 -2.458 -2.009 |
| 48 omega.b 0.785 0.092 0.774 1.00 5.0 0.654 0.952 |
| 49 sigma.b[1] 0.991 0.587 0.698 0.99 5.0 0.450 1.909 |
| 50 sigma.b[2] 0.846 0.599 0.556 0.99 4.6 0.372 1.690 |
| 51 sigma.b[3] 0.768 0.503 0.556 0.99 5.0 0.361 1.500 |
| 52 sigma.b[4] 0.787 0.424 0.579 1.00 5.0 0.366 1.595 |
| 53 sigma.b[5] 0.799 0.667 0.546 1.02 5.0 0.354 1.615 |
| 54 sigma.b[6] 0.809 0.523 0.530 0.99 5.0 0.356 1.821 |
| 55 sigma.b[7] 1.077 1.492 0.615 1.00 5.0 0.448 2.191 |
| 56 sigma.b[8] 0.961 0.775 0.648 0.99 5.0 0.405 2.413 |
| 57 sigma.b[9] 0.856 0.470 0.587 0.99 5.0 0.371 1.769 |
| 58 sigma.b[10] 0.802 0.515 0.535 1.00 5.0 0.381 1.701 |
| 59 sigma.b[11] 0.794 0.550 0.547 1.00 5.0 0.344 1.791 |
| 60 sigma.b[12] 0.791 0.511 0.494 1.00 6.2 0.354 1.756 |
| 61 sigma.b[13] 0.775 0.475 0.516 0.99 5.0 0.345 1.645 |
| 62 sigma.b[14] 0.770 0.455 0.514 1.02 5.0 0.369 1.608 |
| 63 sigma.b[15] 0.812 0.558 0.500 1.00 5.0 0.384 1.660 |
| 64 sigma.b[16] 0.789 0.547 0.547 1.00 5.6 0.353 1.562 |
| 65 sigma.b[17] 0.753 0.408 0.525 1.00 5.0 0.353 1.547 |
| 66 sigma.b[18] 0.803 0.530 0.516 1.00 5.0 0.358 1.860 |
| 67 sigma.b[19] 0.856 0.730 0.592 1.00 5.0 0.365 1.625 |
| 68 sigma.b[20] 0.801 0.537 0.547 1.00 5.0 0.370 1.720 |
| 69 sigma.b[21] 0.811 0.570 0.577 1.00 5.0 0.361 1.868 |
| 70 sigma.b[22] 0.785 0.475 0.489 0.99 4.8 0.352 1.570 |
| 71 sigma.b[23] 0.842 0.622 0.540 0.99 5.0 0.364 1.882 |
| 72 sigma.b[24] 0.770 0.451 0.536 0.99 5.0 0.374 1.517 |
| 73 sigma.b[25] 0.812 0.560 0.556 0.99 5.0 0.385 1.623 |
| 74 sigma.b[26] 0.823 0.549 0.551 1.00 5.0 0.358 1.885 |
| 75 sigma.b[27] 0.811 0.473 0.554 1.00 5.0 0.375 1.653 |
| 76 sigma.b[28] 0.752 0.475 0.500 0.99 5.0 0.361 1.491 |
| 77 sigma.b[29] 0.787 0.548 0.517 0.99 4.7 0.342 1.671 |
| 78 sigma.b[30] 0.779 0.443 0.517 1.00 5.0 0.348 1.656 |
| 79 sigma.b[31] 0.819 0.606 0.490 1.02 5.0 0.359 1.689 |
| 80 sigma.b[32] 0.757 0.420 0.519 0.99 5.0 0.375 1.617 |
| 81 sigma.b[33] 0.812 0.724 0.576 1.00 5.0 0.351 1.857 |
| 82 sigma.b[34] 0.813 0.690 0.530 1.01 5.0 0.352 1.622 |
| 83 sigma.b[35] 0.813 0.497 0.604 1.00 5.0 0.376 1.630 |
| 84 sigma.b[36] 0.811 0.554 0.508 1.00 5.0 0.364 1.741 |
| 85 sigma.b[37] 0.841 0.604 0.538 1.00 5.0 0.370 1.907 |
| 86 sigma.b[38] 0.768 0.406 0.570 1.01 5.4 0.370 1.630 |
| 87 sigma.b[39] 0.830 0.623 0.566 1.00 4.6 0.344 1.851 |
| 88 sigma.b[40] 0.767 0.556 0.499 1.00 5.5 0.347 1.582 |
| 89 sigma.b[41] 0.777 0.440 0.482 1.00 5.0 0.369 1.767 |
| 90 sigma.b[42] 0.838 0.517 0.533 1.00 5.3 0.389 1.803 |
| 91 sigma.res[1] 0.807 0.031 0.806 1.00 5.0 0.759 0.862 |
| 92 sigma.res[2] 0.904 0.035 0.909 0.99 5.4 0.847 0.961 |
| 93 sigma.res[3] 1.250 0.049 1.239 0.99 5.0 1.177 1.332 |
| 94 sigma.res[4] 0.963 0.039 0.950 1.01 4.0 0.904 1.033 |
| 95 sigma.res[5] 1.075 0.039 1.077 1.00 5.0 1.011 1.145 |
| 96 sigma.res[6] 0.900 0.035 0.885 1.02 4.6 0.843 0.958 |
| 97 sigma.res[7] 0.808 0.030 0.801 0.99 5.0 0.762 0.852 |
| 98 sigma.res[8] 1.739 0.067 1.740 0.99 5.0 1.627 1.852 |
| 99 sigma.res[9] 1.847 0.072 1.839 1.00 5.0 1.727 1.966 |
| 100 sigma.res[10] 0.362 0.015 0.360 1.00 5.4 0.338 0.386 |
| 101 sigma.res[11] 0.342 0.013 0.341 1.00 5.5 0.322 0.365 |
| 102 sigma.res[12] 0.411 0.016 0.408 0.99 4.6 0.384 0.437 |
| 103 sigma.res[13] 0.355 0.014 0.358 1.02 5.0 0.333 0.379 |
| 104 sigma.res[14] 0.349 0.013 0.345 1.01 5.6 0.328 0.373 |
| 105 sigma.res[15] 0.301 0.013 0.297 0.99 6.0 0.281 0.323 |
| 106 sigma.res[16] 0.321 0.013 0.321 1.00 5.4 0.300 0.343 |
| 107 sigma.res[17] 0.308 0.013 0.307 1.00 5.8 0.286 0.329 |
| 108 sigma.res[18] 0.344 0.015 0.344 0.99 5.5 0.321 0.369 |
| 109 sigma.res[19] 0.315 0.012 0.312 1.05 5.9 0.295 0.336 |
| 110 sigma.res[20] 0.356 0.015 0.355 0.99 5.0 0.331 0.382 |
| 111 sigma.res[21] 0.393 0.016 0.389 0.99 5.0 0.369 0.423 |
| 112 sigma.res[22] 0.367 0.015 0.368 1.00 5.0 0.343 0.391 |
| 113 sigma.res[23] 0.392 0.015 0.392 1.01 4.8 0.368 0.415 |
| 114 sigma.res[24] 0.337 0.013 0.337 1.00 5.5 0.315 0.359 |
| 115 sigma.res[25] 0.317 0.013 0.315 1.01 6.6 0.296 0.340 |
| 116 sigma.res[26] 0.339 0.014 0.338 1.01 5.6 0.318 0.364 |
| 117 sigma.res[27] 0.332 0.014 0.332 1.00 5.0 0.311 0.357 |
| 118 sigma.res[28] 0.362 0.015 0.357 1.00 5.0 0.339 0.389 |
| 119 sigma.res[29] 0.344 0.015 0.341 0.99 5.4 0.319 0.370 |
| 120 sigma.res[30] 0.336 0.013 0.335 1.00 6.3 0.315 0.360 |
| 121 sigma.res[31] 0.357 0.015 0.361 1.00 5.0 0.334 0.383 |
| 122 sigma.res[32] 0.657 0.025 0.656 1.00 5.0 0.620 0.700 |
| 123 sigma.res[33] 0.499 0.020 0.498 1.00 5.0 0.468 0.531 |
| 124 sigma.res[34] 0.372 0.015 0.374 1.00 5.0 0.348 0.398 |
| 125 sigma.res[35] 0.333 0.013 0.328 0.99 5.0 0.312 0.355 |
| 126 sigma.res[36] 0.633 0.024 0.634 1.01 5.0 0.593 0.672 |
| 127 sigma.res[37] 0.350 0.014 0.348 0.99 5.0 0.327 0.374 |
| 128 sigma.res[38] 0.384 0.015 0.373 1.01 6.5 0.361 0.410 |
| 129 sigma.res[39] 0.337 0.013 0.337 1.00 5.4 0.316 0.360 |
| 130 sigma.res[40] 0.344 0.014 0.345 1.00 4.7 0.323 0.368 |
| 131 sigma.res[41] 0.520 0.020 0.517 1.00 5.0 0.489 0.553 |
| 132 sigma.res[42] 0.450 0.019 0.446 1.02 5.5 0.421 0.481 |
