# Supplementary material for: Data on motivational factors of the medical and nursing staff of a Greek Public Regional General Hospital during the economic crisis
Source: Data Brief. 2017 Feb 16;11:371–81. doi: 10.1016/j.dib.2017.02.026 (PMC5331154; doi:10.1016/j.dib.2017.02.026)
Supplement: Supplementary file 2 — Supplementary material [file mmc2.zip › Model 1.docx]

Table 5

Model 1

Model without intercept variances and no slopes.

| Item Parameters |
| --- |
| parameter Mean SD MAP Rhat PercSEratio Q5 Q95 |
| 1 deviance 9592.034 14.337 9592.378 1.00 5.0 9569.364 9615.766 |
| 2 b[1] -1.650 0.053 -1.642 0.99 5.0 -1.737 -1.567 |
| 3 b[2] -1.692 0.050 -1.706 1.00 5.0 -1.770 -1.612 |
| 4 b[3] -2.596 0.068 -2.606 1.00 5.0 -2.708 -2.482 |
| 5 b[4] -2.092 0.050 -2.098 0.99 5.0 -2.179 -2.007 |
| 6 b[5] -1.845 0.060 -1.857 1.00 5.0 -1.934 -1.747 |
| 7 b[6] -1.241 0.048 -1.242 1.00 5.0 -1.322 -1.162 |
| 8 b[7] -1.648 0.052 -1.644 1.00 5.0 -1.729 -1.569 |
| 9 b[8] -2.527 0.097 -2.525 1.01 4.3 -2.681 -2.360 |
| 10 b[9] -3.236 0.098 -3.252 1.00 5.0 -3.408 -3.073 |
| 11 b[10] -1.636 0.019 -1.635 1.00 5.0 -1.667 -1.605 |
| 12 b[11] -1.851 0.019 -1.850 1.04 5.0 -1.883 -1.822 |
| 13 b[12] -1.746 0.022 -1.741 0.99 4.2 -1.781 -1.709 |
| 14 b[13] -2.018 0.019 -2.018 0.99 5.0 -2.052 -1.988 |
| 15 b[14] -2.028 0.019 -2.027 0.99 5.0 -2.059 -1.997 |
| 16 b[15] -2.600 0.016 -2.598 0.99 5.0 -2.626 -2.573 |
| 17 b[16] -2.725 0.018 -2.727 1.00 5.0 -2.755 -2.695 |
| 18 b[17] -2.642 0.018 -2.638 1.00 5.0 -2.672 -2.611 |
| 19 b[18] -2.364 0.019 -2.373 0.99 5.0 -2.394 -2.331 |
| 20 b[19] -2.212 0.020 -2.216 0.99 5.0 -2.244 -2.179 |
| 21 b[20] -2.054 0.019 -2.055 1.00 5.0 -2.085 -2.023 |
| 22 b[21] -3.085 0.023 -3.088 1.01 5.5 -3.123 -3.048 |
| 23 b[22] -3.249 0.021 -3.251 0.99 5.0 -3.285 -3.216 |
| 24 b[23] -3.018 0.021 -3.017 1.00 5.0 -3.054 -2.986 |
| 25 b[24] -3.080 0.018 -3.073 1.01 5.0 -3.109 -3.051 |
| 26 b[25] -3.177 0.018 -3.180 1.00 4.6 -3.207 -3.149 |
| 27 b[26] -3.321 0.018 -3.321 1.00 5.0 -3.352 -3.293 |
| 28 b[27] -3.258 0.018 -3.258 0.99 5.0 -3.288 -3.228 |
| 29 b[28] -3.327 0.021 -3.319 1.00 4.4 -3.361 -3.294 |
| 30 b[29] -3.245 0.019 -3.240 1.00 5.0 -3.277 -3.213 |
| 31 b[30] -3.306 0.018 -3.304 0.99 5.6 -3.335 -3.277 |
| 32 b[31] -3.131 0.019 -3.126 0.99 5.0 -3.161 -3.100 |
| 33 b[32] -0.528 0.036 -0.526 0.99 5.6 -0.587 -0.469 |
| 34 b[33] -1.028 0.026 -1.030 0.99 5.0 -1.073 -0.987 |
| 35 b[34] -1.318 0.023 -1.321 1.00 5.0 -1.356 -1.281 |
| 36 b[35] -1.658 0.019 -1.660 0.99 4.6 -1.689 -1.629 |
| 37 b[36] -1.050 0.035 -1.047 1.00 5.0 -1.106 -0.992 |
| 38 b[37] -2.019 0.017 -2.022 1.00 4.5 -2.047 -1.992 |
| 39 b[38] -2.086 0.020 -2.086 0.99 5.0 -2.119 -2.053 |
| 40 b[39] -2.090 0.019 -2.091 1.00 5.0 -2.123 -2.061 |
| 41 b[40] -1.932 0.020 -1.937 1.01 5.6 -1.963 -1.900 |
| 42 b[41] -1.212 0.030 -1.213 1.00 5.0 -1.262 -1.165 |
| 43 b[42] -1.410 0.027 -1.415 1.00 5.0 -1.451 -1.364 |
| 44 sigma1 0.807 0.032 0.805 1.00 5.8 0.755 0.861 |
| 45 sigma2 0.316 0.183 0.215 0.99 5.0 0.147 0.652 |
| 46 ICC 0.139 0.124 0.064 0.99 5.0 0.033 0.405 |
| 47 mu.b -2.241 0.120 -2.260 0.99 5.0 -2.434 -2.054 |
| 48 omega.b 0.779 0.086 0.742 1.01 5.0 0.645 0.920 |
| 49 sigma.res[1] 0.929 0.037 0.927 1.02 5.6 0.874 0.997 |
| 50 sigma.res[2] 0.912 0.037 0.908 1.00 5.0 0.854 0.975 |
| 51 sigma.res[3] 1.253 0.049 1.249 1.00 5.0 1.178 1.342 |
| 52 sigma.res[4] 0.968 0.035 0.954 0.99 4.4 0.915 1.032 |
| 53 sigma.res[5] 1.077 0.041 1.073 1.00 5.0 1.008 1.144 |
| 54 sigma.res[6] 0.903 0.035 0.893 1.00 5.0 0.851 0.957 |
| 55 sigma.res[7] 0.929 0.036 0.926 1.00 4.8 0.875 0.992 |
| 56 sigma.res[8] 1.773 0.076 1.772 1.01 5.0 1.656 1.897 |
| 57 sigma.res[9] 1.855 0.076 1.840 0.99 5.0 1.738 1.992 |
| 58 sigma.res[10] 0.370 0.015 0.365 0.99 5.0 0.347 0.395 |
| 59 sigma.res[11] 0.349 0.015 0.349 0.99 5.6 0.326 0.373 |
| 60 sigma.res[12] 0.415 0.016 0.415 1.00 5.7 0.389 0.441 |
| 61 sigma.res[13] 0.357 0.014 0.358 1.01 4.6 0.335 0.379 |
| 62 sigma.res[14] 0.351 0.015 0.354 1.00 6.0 0.328 0.375 |
| 63 sigma.res[15] 0.301 0.012 0.301 1.00 6.0 0.281 0.320 |
| 6 sigma.res[16] 0.320 0.012 0.320 1.00 5.7 0.301 0.339 |
| 65 sigma.res[17] 0.310 0.013 0.309 1.00 5.5 0.292 0.333 |
| 66 sigma.res[18] 0.344 0.014 0.345 1.00 5.4 0.322 0.368 |
| 67 sigma.res[19] 0.366 0.015 0.363 1.00 5.7 0.342 0.393 |
| 68 sigma.res[20] 0.366 0.014 0.364 0.99 5.0 0.342 0.391 |
| 69 sigma.res[21] 0.395 0.016 0.392 0.99 5.8 0.371 0.423 |
| 70 sigma.res[22] 0.364 0.015 0.367 1.00 6.1 0.340 0.388 |
| 71 sigma.res[23] 0.387 0.016 0.391 0.99 5.4 0.363 0.413 |
| 72 sigma.res[24] 0.334 0.014 0.328 0.99 6.3 0.313 0.357 |
| 73 sigma.res[25] 0.325 0.013 0.324 0.99 5.4 0.304 0.346 |
| 74 sigma.res[26] 0.335 0.014 0.336 1.00 5.0 0.312 0.362 |
| 75 sigma.res[27] 0.331 0.014 0.328 1.00 5.6 0.309 0.355 |
| 76 sigma.res[28] 0.359 0.014 0.362 0.99 5.0 0.338 0.383 |
| 77 sigma.res[29] 0.341 0.013 0.343 0.99 6.5 0.319 0.362 |
| 78 sigma.res[30] 0.332 0.014 0.331 0.99 5.7 0.310 0.355 |
| 79 sigma.res[31] 0.358 0.015 0.356 1.00 6.4 0.336 0.382 |
| 80 sigma.res[32] 0.666 0.025 0.665 1.01 5.0 0.625 0.706 |
| 81 sigma.res[33] 0.501 0.020 0.505 0.99 5.8 0.470 0.535 |
| 82 sigma.res[34] 0.395 0.016 0.399 1.00 5.5 0.370 0.424 |
| 83 sigma.res[35] 0.339 0.015 0.337 0.99 5.0 0.316 0.366 |
| 84 sigma.res[36] 0.640 0.025 0.636 1.00 5.0 0.601 0.684 |
| 85 sigma.res[37] 0.356 0.014 0.357 0.99 5.0 0.333 0.380 |
| 86 sigma.res[38] 0.391 0.016 0.387 0.99 5.8 0.366 0.418 |
| 87 sigma.res[39] 0.339 0.014 0.337 0.99 5.8 0.319 0.362 |
| 88 sigma.res[40] 0.358 0.015 0.356 1.00 5.0 0.335 0.384 |
| 89 sigma.res[41] 0.538 0.022 0.539 0.99 5.0 0.502 0.573 |
| 90 sigma.res[42] 0.512 0.020 0.515 1.00 5.0 0.478 0.545 |
