# Supplementary material for: Data on motivational factors of the medical and nursing staff of a Greek Public Regional General Hospital during the economic crisis
Source: Data Brief. 2017 Feb 16;11:371–81. doi: 10.1016/j.dib.2017.02.026 (PMC5331154; doi:10.1016/j.dib.2017.02.026)
Supplement: Supplementary file 2 — Supplementary material [file mmc2.zip › Model 3.docx]

Table 7

Model 3

Model with intercept variance and slope variances with hierarchical item and slope parameters.

| Item Parameters |
| --- |
| parameter Mean SD MAP Rhat PercSEratio Q5 Q95 |
| 1 deviance 5208.510 315.575 5371.633 1.00 5.0 4663.664 5646.900 |
| 2 b[1] -1.500 0.001 -1.500 0.99 5.0 -1.502 -1.498 |
| 3 b[2] -1.642 0.026 -1.641 1.00 5.0 -1.686 -1.599 |
| 4 b[3] -2.618 0.048 -2.613 0.99 5.0 -2.698 -2.545 |
| 5 b[4] -2.129 0.030 -2.129 1.00 5.0 -2.175 -2.083 |
| 6 b[5] -1.820 0.041 -1.827 1.00 5.0 -1.886 -1.753 |
| 7 b[6] -1.227 0.026 -1.227 0.99 5.0 -1.267 -1.185 |
| 8 b[7] -1.500 0.002 -1.500 0.99 5.5 -1.503 -1.497 |
| 9 b[8] -2.406 0.081 -2.392 0.99 4.5 -2.539 -2.273 |
| 10 b[9] -3.150 0.085 -3.121 1.00 5.0 -3.289 -3.013 |
| 11 b[10] -1.611 0.022 -1.612 1.13 9.0 -1.647 -1.575 |
| 12 b[11] -1.829 0.020 -1.830 1.21 14.4 -1.862 -1.795 |
| 13 b[12] -1.758 0.019 -1.760 1.07 8.6 -1.789 -1.728 |
| 14 b[13] -2.021 0.019 -2.013 1.15 9.1 -2.051 -1.989 |
| 15 b[14] -2.019 0.020 -2.022 1.16 15.1 -2.050 -1.986 |
| 16 b[15] -2.590 0.019 -2.600 1.17 18.3 -2.619 -2.559 |
| 17 b[16] -2.713 0.020 -2.724 1.24 10.0 -2.742 -2.680 |
| 18 b[17] -2.624 0.019 -2.633 1.11 11.5 -2.652 -2.591 |
| 19 b[18] -2.356 0.019 -2.354 1.14 7.4 -2.388 -2.325 |
| 20 b[19] -2.153 0.020 -2.151 1.08 5.9 -2.187 -2.122 |
| 21 b[20] -2.030 0.019 -2.030 1.15 10.4 -2.061 -2.001 |
| 22 b[21] -3.065 0.023 -3.067 1.08 9.5 -3.103 -3.026 |
| 23 b[22] -3.255 0.024 -3.252 1.05 7.6 -3.292 -3.218 |
| 24 b[23] -3.017 0.024 -3.015 1.06 10.1 -3.057 -2.978 |
| 25 b[24] -3.085 0.023 -3.084 1.14 15.9 -3.120 -3.047 |
| 26 b[25] -3.203 0.020 -3.205 1.07 7.1 -3.237 -3.168 |
| 27 b[26] -3.317 0.020 -3.317 1.08 7.3 -3.352 -3.286 |
| 28 b[27] -3.242 0.021 -3.248 1.21 17.7 -3.274 -3.209 |
| 29 b[28] -3.318 0.023 -3.321 1.10 8.8 -3.355 -3.280 |
| 30 b[29] -3.254 0.021 -3.256 1.08 10.4 -3.287 -3.221 |
| 31 b[30] -3.301 0.020 -3.297 1.04 8.4 -3.335 -3.268 |
| 32 b[31] -3.141 0.024 -3.147 1.11 8.3 -3.179 -3.098 |
| 33 b[32] -0.550 0.037 -0.541 1.11 9.1 -0.612 -0.494 |
| 34 b[33] -1.031 0.028 -1.031 1.11 12.9 -1.078 -0.988 |
| 35 b[34] -1.352 0.023 -1.348 1.16 11.5 -1.390 -1.315 |
| 36 b[35] -1.673 0.021 -1.678 1.09 15.3 -1.709 1.641 |
| 37 b[36] -1.077 0.037 -1.082 1.11 8.5 -1.136 -1.013 |
| 38 b[37] -2.029 0.022 -2.030 1.14 10.4 -2.065 -1.993 |
| 39 b[38] -2.103 0.024 -2.096 1.13 13.4 -2.143 -2.065 |
| 40 b[39] -2.082 0.019 -2.079 1.09 8.0 -2.114 -2.050 |
| 41 b[40] -1.954 0.019 -1.957 1.06 5.5 -1.985 -1.922 |
| 42 b[41] -1.249 0.028 -1.249 1.09 9.8 -1.297 -1.204 |
| 43 b[42] -1.481 0.025 -1.491 1.09 8.5 -1.519 -1.436 |
| 44 a[1] -0.015 0.014 -0.020 0.99 5.0 -0.039 0.009 |
| 45 a[2] 0.011 0.049 0.019 1.00 4.3 -0.078 0.089 |
| 46 a[3] -0.153 0.073 -0.132 1.00 5.0 -0.271 -0.028 |
| 47 a[4] -0.019 0.050 -0.020 1.02 5.5 -0.106 0.059 |
| 48 a[5] -0.029 0.084 -0.018 1.01 5.0 -0.163 0.113 |
| 49 a[6] 0.065 0.040 0.064 1.02 5.0 0.001 0.128 |
| 50 a[7] -0.015 0.018 -0.013 1.00 5.0 -0.045 0.011 |
| 51 a[8] -0.157 0.121 -0.183 1.01 5.0 -0.353 0.031 |
| 52 a[9] -0.231 0.127 -0.219 1.01 5.0 -0.425 -0.027 |
| 53 a[10] 1.314 0.032 1.322 1.00 4.2 1.261 1.364 |
| 54 a[11] 1.408 0.032 1.407 1.03 5.6 1.355 1.458 |
| 55 a[12] 1.461 0.034 1.457 1.00 5.5 1.403 1.516 |
| 56 a[13] 1.436 0.033 1.426 0.99 5.0 1.383 1.492 |
| 57 a[14] 1.402 0.030 1.408 1.01 5.0 1.355 1.451 |
| 58 a[15] 1.270 0.030 1.275 1.03 5.5 1.221 1.322 |
| 59 a[16] 1.343 0.031 1.332 1.00 5.0 1.292 1.391 |
| 60 a[17] 1.274 0.031 1.278 1.01 5.0 1.224 1.326 |
| 61 a[18] 1.398 0.032 1.406 0.99 6.3 1.346 1.446 |
| 62 a[19] 1.222 0.032 1.224 1.01 5.0 1.170 1.276 |
| 63 a[20] 1.399 0.029 1.398 0.99 5.0 1.351 1.447 |
| 64 a[21] 1.399 0.038 1.413 0.99 5.0 1.330 1.465 |
| 65 a[22] 1.229 0.033 1.234 0.99 5.0 1.177 1.284 |
| 66 a[23] 1.450 0.050 1.457 1.00 5.0 1.369 1.532 |
| 67 a[24] 1.293 0.043 1.289 1.00 5.0 1.223 1.368 |
| 68 a[25] 1.172 0.043 1.187 1.00 5.0 1.103 1.249 |
| 69 a[26] 0.982 0.071 0.973 1.00 5.0 0.867 1.091 |
| 70 a[27] 1.129 0.032 1.135 1.01 5.6 1.075 1.185 |
| 71 a[28] 1.091 0.033 1.079 0.99 5.4 1.036 1.146 |
| 72 a[29] 1.106 0.032 1.099 1.00 5.0 1.050 1.156 |
| 73 a[30] 1.100 0.032 1.103 1.00 5.0 1.051 1.153 |
| 74 a[31] 1.290 0.034 1.297 1.02 5.0 1.231 1.348 |
| 75 a[32] 1.106 0.054 1.092 1.00 3.7 1.012 1.191 |
| 76 a[33] 1.434 0.042 1.435 0.99 5.0 1.365 1.502 |
| 77 a[34] 1.407 0.033 1.415 1.00 5.8 1.354 1.462 |
| 78 a[35] 1.317 0.029 1.316 1.00 5.0 1.270 1.368 |
| 79 a[36] 1.617 0.048 1.611 0.99 6.0 1.536 1.687 |
| 80 a[37] 1.210 0.035 1.213 1.00 5.0 1.151 1.268 |
| 81 a[38] 1.272 0.034 1.282 1.01 5.0 1.214 1.326 |
| 82 a[39] 1.098 0.034 1.094 1.00 5.0 1.045 1.149 |
| 83 a[40] 1.103 0.035 1.105 1.00 5.0 1.049 1.161 |
| 84 a[41] 1.530 0.038 1.529 1.00 5.5 1.465 1.591 |
| 85 a[42] 1.282 0.038 1.280 1.00 5.0 1.217 1.339 |
| 86 sigma1 0.679 0.029 0.672 1.02 8.8 0.632 0.726 |
| 87 sigma2 0.321 0.188 0.234 1.00 5.0 0.151 0.711 |
| 88 ICC 0.182 0.147 0.095 1.00 5.0 0.047 0.522 |
| 89 mu.b -2.227 0.119 -2.210 1.00 5.0 -2.426 - 2.044 |
| 90 omega.b 0.781 0.090 0.804 1 .00 5.0 0.640 0.938 |
| 91 sigma.b[1] 0.954 0.582 0.649 0.99 5.0 0.423 1.791 |
| 92 sigma.b[2] 0.810 0.519 0.565 1.00 5.0 0.373 1.690 |
| 93 sigma.b[3] 0.797 0.463 0.544 1.00 3.9 0.353 1.701 |
| 94 sigma.b[4] 0.784 0.466 0.537 1.01 5.9 0.360 1.733 |
| 95 sigma.b[5] 0.810 0.573 0.551 1.00 5.0 0.381 1.854 |
| 96 sigma.b[6] 0.783 0.530 0.537 1.01 5.0 0.342 1.574 |
| 97 sigma.b[7] 0.924 0.574 0.652 1.00 5.0 0.426 1.931 |
| 98 sigma.b[8] 0.910 0.617 0.614 1.03 5.0 0.412 2.152 |
| 99 sigma.b[9] 0.834 0.512 0.536 0.99 5.2 0.386 1.801 |
| 100 sigma.b[10] 0.791 0.789 0.538 1.00 5.0 0.367 1.495 |
| 101 sigma.b[11] 0.861 0.602 0.602 1.00 5.0 0.378 1.939 |
| 102 sigma.b[12] 0.741 0.427 0.534 0.99 4.6 0.373 1.378 |
| 103 sigma.b[13] 0.818 0.612 0.547 1.01 5.0 0.350 1.784 |
| 104 sigma.b[14] 0.783 0.604 0.489 1.01 5.0 0.351 1.601 |
| 105 sigma.b[15] 0.819 0.840 0.560 0.99 5.0 0.362 1.657 |
| 106 sigma.b[16] 0.793 0.558 0.519 1.00 5.0 0.366 1.630 |
| 107 sigma.b[17] 0.822 0.570 0.522 1.00 5.0 0.360 1.815 |
| 108 sigma.b[18] 0.754 0.451 0.529 0.99 5.0 0.361 1.583 |
| 109 sigma.b[19] 0.863 0.747 0.569 1.00 5.0 0.369 1.735 |
| 110 sigma.b[20] 0.815 0.624 0.529 1.00 4.3 0.355 1.694 |
| 111 sigma.b[21] 0.782 0.462 0.581 1.01 5.0 0.371 1.533 |
| 112 sigma.b[22] 0.799 0.447 0.541 1.00 5.0 0.354 1.628 |
| 113 sigma.b[23] 0.766 0.443 0.514 1.01 5.0 0.339 1.425 |
| 114 sigma.b[24] 0.764 0.457 0.528 0.99 5.0 0.352 1.703 |
| 115 sigma.b[25] 0.809 0.539 0.543 1.01 5.0 0.379 1.762 |
| 116 sigma.b[26] 0.802 0.538 0.536 1.00 5.6 0.375 1.668 |
| 117 sigma.b[27] 0.823 0.514 0.555 1.01 5.0 0.357 1.726 |
| 118 sigma.b[28] 0.785 0.512 0.518 1.00 5.0 0.371 1.674 |
| 119 sigma.b[29] 0.803 0.520 0.538 1.00 5.0 0.372 1.699 |
| 120 sigma.b[30] 0.872 1.017 0.525 1.00 5.0 0.352 2.064 |
| 121 sigma.b[31] 0.839 0.793 0.522 1.00 5.4 0.345 1.679 |
| 122 sigma.b[32] 0.825 0.655 0.52 1.00 5.0 0.355 1.910 |
| 123 sigma.b[33] 0.793 0.484 0.549 1.00 5.0 0.370 1.660 |
| 124 sigma.b[34] 0.815 0.544 0.536 1.02 4.6 0.380 1.651 |
| 125 sigma.b[35] 0.807 0.528 0.542 1.00 5.0 0.358 1.800 |
| 126 sigma.b[36] 0.777 0.434 0.522 1.00 4.6 0.367 1.625 |
| 127 sigma.b[37] 0.812 0.497 0.532 1.00 5.0 0.367 1.770 |
| 128 sigma.b[38] 0.788 0.506 0.550 1.00 5.0 0.358 1.618 |
| 129 sigma.b[39] 0.744 0.391 0.530 1.00 5.0 0.354 1.533 |
| 130 sigma.b[40] 0.800 0.628 0.560 1.00 5.0 0.343 1.735 |
| 131 sigma.b[41] 0.822 0.563 0.541 1.01 5.0 0.365 1.738 |
| 132 sigma.b[42] 0.821 0.499 0.533 0.99 5.4 0.392 1.779 |
| 133 sigma.a[1] 0.811 0.591 0.523 0.99 5.0 0.359 1.708 |
| 134 sigma.a[2] 0.759 0.425 0.598 0.99 4.2 0.339 1.688 |
| 135 sigma.a[3] 0.765 0.527 0.510 1.00 5.0 0.372 1.586 |
| 136 sigma.a[4] 0.823 0.637 0.522 1.00 5.0 0.356 1.966 |
| 137 sigma.a[5] 0.853 0.578 0.552 0.99 5.5 0.367 1.783 |
| 138 sigma.a[6] 0.792 0.585 0.538 1.01 6.6 0.361 1.542 |
| 139 sigma.a[7] 0.826 0.674 0.543 1.00 5.1 0.342 1.794 |
| 140 sigma.a[8] 0.802 0.489 0.522 1.00 5.0 0.356 1.660 |
| 141 sigma.a[9] 0.809 0.618 0.562 1.00 4.6 0.381 1.620 |
| 142 sigma.a[10] 0.805 0.499 0.520 1.00 5.0 0.375 1.636 |
| 143 sigma.a[11] 0.848 0.541 0.616 1.00 5.0 0.384 1.964 |
| 144 sigma.a[12] 0.806 0.599 0.556 1.00 5.0 0.360 1.798 |
| 145 sigma.a[13] 0.827 0.547 0.519 0.99 5.0 0.383 1.860 |
| 146 sigma.a[14] 0.836 0.800 0.515 1.00 5.0 0.371 1.842 |
| 147 sigma.a[15] 0.819 0.489 0.549 1.00 5.0 0.378 1.794 |
| 148 sigma.a[16] 0.852 0.617 0.565 1.00 5.0 0.358 1.746 |
| 149 sigma.a[17] 0.807 0.568 0.544 1.02 5.0 0.358 1.632 |
| 150 sigma.a[18] 0.807 0.521 0.550 1.00 5.0 0.359 1.731 |
| 151 sigma.a[19] 0.848 0.676 0.552 1.03 5.0 0.363 1.856 |
| 152 sigma.a[20] 0.811 0.560 0.492 1.00 5.4 0.366 1.753 |
| 153 sigma.a[21] 0.900 0.765 0.550 0.99 5.0 0.379 2.027 |
| 154 sigma.a[22] 0.799 0.455 0.513 1.00 4.4 0.362 1.641 |
| 155 sigma.a[23] 0.840 0.567 0.553 0.99 4.8 0.380 1.875 |
| 156 sigma.a[24] 0.798 0.424 0.564 0.99 4.2 0.371 1.693 |
| 157 sigma.a[25] 0.788 0.444 0.527 1.00 5.0 0.363 1.700 |
| 158 sigma.a[26] 0.800 0.659 0.571 0.99 5.8 0.359 1.651 |
| 159 sigma.a[27] 0.840 0.584 0.589 0.99 5.0 0.381 1.643 |
| 160 sigma.a[28] 0.810 0.528 0.523 1.00 5.0 0.383 1.855 |
| 161 sigma.a[29] 0.821 0.506 0.558 1.00 6.4 0.351 1.774 |
| 162 sigma.a[30] 0.799 0.538 0.533 1.00 5.0 0.366 1.942 |
| 163 sigma.a[31] 0.784 0.524 0.501 1.00 5.0 0.336 1.600 |
| 164 sigma.a[32] 0.872 0.565 0.585 1.00 5.0 0.378 1.991 |
| 165 sigma.a[33] 0.813 0.530 0.544 0.99 5.0 0.382 1.629 |
| 166 sigma.a[34] 0.841 0.577 0.555 1.00 5.0 0.379 2.039 |
| 167 sigma.a[35] 0.786 0.493 0.563 1.02 5.0 0.367 1.633 |
| 168 sigma.a[36] 0.816 0.519 0.585 0.99 5.0 0.359 1.670 |
| 169 sigma.a[37] 0.805 0.522 0.522 1.00 4.6 0.359 1.870 |
| 170 sigma.a[38] 0.824 0.606 0.551 1.00 6.4 0.374 1.652 |
| 171 sigma.a[39] 0.861 0.742 0.537 0.99 5.4 0.367 1.930 |
| 172 sigma.a[40] 0.813 0.560 0.547 1.00 5.0 0.347 1.758 |
| 173 sigma.a[41] 0.803 0.492 0.518 1.01 5.9 0.384 1.660 |
| 174 sigma.a[42] 0.801 0.595 0.516 1.00 5.0 0.344 1.944 |
| 175 omega.a 0.581 0.062 0.567 1.01 5.0 0.483 0.688 |
| 176 sigma.res[1] 0.017 0.009 0.014 1.00 5.0 0.005 0.034 |
| 177 sigma.res[2] 0.441 0.018 0.444 1.01 5.0 0.412 0.470 |
| 178 sigma.res[3] 0.865 0.035 0.866 0.99 5.0 0.812 0.920 |
| 179 sigma.res[4] 0.517 0.021 0.514 1.00 5.6 0.482 0.553 |
| 180 sigma.res[5] 0.708 0.029 0.714 1.00 5.0 0.660 0.756 |
| 181 sigma.res[6] 0.467 0.018 0.460 1.00 5.1 0.436 0.498 |
| 182 sigma.res[7] 0.023 0.013 0.018 1.00 5.6 0.006 0.046 |
| 183 sigma.res[8] 1.485 0.060 1.489 1.04 5.0 1.388 1.583 |
| 184 sigma.res[9] 1.595 0.065 1.581 1.00 4.4 1.495 1.711 |
| 185 sigma.res[10] 0.350 0.015 0.348 1.00 5.0 0.325 0.374 |
| 186 sigma.res[11] 0.314 0.013 0.314 0.99 5.8 0.291 0.336 |
| 187 sigma.res[12] 0.338 0.014 0.336 1.00 5.0 0.316 0.364 |
| 188 sigma.res[13] 0.316 0.014 0.317 1.01 5.0 0.294 0.339 |
| 189 sigma.res[14] 0.306 0.011 0.305 1.00 5.4 0.289 0.326 |
| 190 sigma.res[15] 0.300 0.013 0.294 1.00 5.9 0.280 0.322 |
| 191 sigma.res[16] 0.311 0.013 0.308 1.00 5.0 0.289 0.333 |
| 192 sigma.res[17] 0.307 0.013 0.304 1.04 7.7 0.286 0.329 |
| 193 sigma.res[18] 0.293 0.012 0.294 1.00 4.6 0.274 0.313 |
| 194 sigma.res[19] 0.307 0.012 0.306 1.00 4.2 0.291 0.326 |
| 195 sigma.res[20] 0.305 0.013 0.305 1.00 5.6 0.282 0.325 |
| 196 sigma.res[21] 0.401 0.017 0.404 1.00 5.0 0.372 0.430 |
| 197 sigma.res[22] 0.387 0.015 0.379 1.02 5.0 0.365 0.410 |
| 198 sigma.res[23] 0.386 0.016 0.381 1.01 5.7 0.360 0.414 |
| 199 sigma.res[24] 0.352 0.015 0.346 1.00 6.4 0.329 0.377 |
| 200 sigma.res[25] 0.335 0.015 0.333 0.99 5.0 0.314 0.361 |
| 201 sigma.res[26] 0.337 0.021 0.335 1.00 6.9 0.308 0.378 |
| 202 sigma.res[27] 0.322 0.013 0.318 1.00 5.0 0.300 0.345 |
| 203 sigma.res[28] 0.370 0.016 0.369 1.00 5.5 0.343 0.395 |
| 204 sigma.res[29] 0.356 0.015 0.354 1.00 5.0 0.334 0.383 |
| 205 sigma.res[30 0.341 0.014 0.340 0.99 5.9 0.320 0.365 |
| 206 sigma.res[31] 0.375 0.015 0.376 1.01 5.0 0.351 0.402 |
| 207 sigma.res[32] 0.616 0.023 0.617 1.01 6.9 0.579 0.660 |
| 208 sigma.res[33] 0.465 0.018 0.461 1.02 6.8 0.437 0.494 |
| 209 sigma.res[34] 0.348 0.015 0.343 0.99 5.0 0.328 0.371 |
| 210 sigma.res[35] 0.323 0.013 0.320 1.00 5.5 0.303 0.343 |
| 211 sigma.res[36] 0.569 0.024 0.563 1.00 5.0 0.529 0.608 |
| 212 sigma.res[37] 0.341 0.014 0.335 1.00 6.0 0.318 0.365 |
| 213 sigma.res[38] 0.374 0.015 0.369 1.01 5.0 0.351 0.398 |
| 214 sigma.res[39] 0.326 0.013 0.320 1.00 5.0 0.305 0.348 |
| 215 sigma.res[40] 0.330 0.014 0.330 1.00 5.5 0.307 0.354 |
| 216 sigma.res[41] 0.462 0.019 0.463 0.99 5.5 0.431 0.499 |
| 217 sigma.res[42] 0.428 0.016 0.425 1.00 5.0 0.402 0.455 |
